# In Vitro Monitoring of Magnesium-Based Implants Degradation by Surface Analysis and Optical Spectroscopy

**DOI:** 10.3390/ijms23116099

**Published:** 2022-05-29

**Authors:** Hafiz Wajahat Hassan, Maryam Rahmati, Alejandro Barrantes, Håvard Jostein Haugen, Peyman Mirtaheri

**Affiliations:** 1 Department of Mechanical, Electronic and Chemical Engineering, Faculty of Technology, Art and Design, Oslo Metropolitan University, 0130 Oslo, Norway; wajahath@oslomet.no; 2 Department of Biomaterials, Institute of Clinical Dentistry and Oral Research Laboratory, University of Oslo, 0317 Oslo, Norway; maryam.rahmati@odont.uio.no (M.R.); a.b.bautista@odont.uio.no (A.B.); h.j.haugen@odont.uio.no (H.J.H.)

**Keywords:** degradation of magnesium, optical spectroscopy, partial-least-squares regression, bubble formation, in vitro monitoring

## Abstract

Magnesium (Mg)-based degradable alloys have attracted substantial attention for tissue engineering applications due to their biodegradability and potential for avoiding secondary removal surgeries. However, insufficient data in the existing literature regarding Mg’s corrosion and gas formation after implantation have delayed its wide clinical application. Since the surface properties of degradable materials constantly change after contact with body fluid, monitoring the behaviour of Mg in phantoms or buffer solutions could provide some information about its physicochemical surface changes over time. Through surface analysis and spectroscopic analysis, we aimed to investigate the structural and functional properties of degradable disks. Since bubble formation may lead to inflammation and change pH, monitoring components related to acidosis near the cells is essential. To study the bubble formation in cell culture media, we used a newly developed Mg alloy (based on Mg, zinc, and calcium), pure Mg, and commercially available grade 2 Titanium (Ti) disks in Dulbecco’s Modified Eagle Medium (DMEM) solution to observe their behaviour over ten days of immersion. Using surface analysis and the information from near-infrared spectroscopy (NIRS), we concluded on the conditions associated with the medical risks of Mg alloy disintegration. NIRS is used to investigate the degradation behaviour of Mg-based disks in the cell culture media, which is correlated with the surface analysis where possible.

## 1. Introduction

Magnesium (Mg)-based alloys are commonly used as structural metals and materials. Over the last few years, their usage as degradable biomaterials has attracted much attention for tissue engineering applications [1]. Mg alloys are suitable for applications such as orthopaedic biomaterials as they have an elastic modulus (~45 GPa) closer to bone, biocompatibility properties, and the ability to promote osteogenesis [2,3,4]. In physiological conditions, Mg and its alloys can degrade and reduce their stiffness over time as the fracture heals, avoiding a second surgery to remove the implant after the bone treatment. This can reduce morbidity rates and costs for patients and healthcare systems [5]. Mg-based implants are bioactive materials that interact with the physiochemical environment and assist the body’s various functions in metabolic reactions, unlike permanent non-degradable Ti implants, which do not take part in any of the metabolic processes inside the body [6,7]. The Mg-based implants are considered as an alternative material over Ti implants because of better bone formation and less inflammatory reactions [8,9]. Compared to Titanium (Ti), Mg-based implants have fewer artefacts when analysing the implant by imaging modalities such as digital radiography (DX), high-resolution flat-panel CT (FPCT), multi-detector computed tomography (MDCT), and magnetic resonance imaging (MRI). All help with post-operative follow-up [10,11]. A variety of plasma electrolytic oxidation (PEO) coating procedures were developed in order to improve corrosion resistance and cytocompatibility and bioactivity of Mg alloys. Additionally, most widely used electrochemical surface treatments for magnesium-based materials were discussed quite recently [12,13]. Another factor highly affecting Mg degradation is the pH change at the bone–implant interface [14]. Thus, in the case of Mg-based implants, due to the active chemical reaction at the implant–tissue interface, hydrogen bubbles are formed, which further may result in the implant’s failure. Hence, it is crucial to examine Mg–fluid interactions precisely before performing any in vivo or clinical trials [15].

Mg research faces the challenge of standardizing in vitro testing procedures compared with in vivo testing methods [16]. Because of their low cost, quick results, and lack of ethical concerns, in vitro tests are very useful. Tests are usually conducted by immersing magnesium samples in a degradation fluid, such as cell culture media, simulated body fluid (SBF), or a 0.9 wt% NaCl solution [3,17]. The bubble formation in vitro and in vivo can be detected by an optical technique called near-infrared spectroscopy (NIRS), which is considered to be a cost-effective and rapid detection method [11]. Although some studies have reported Mg biocompatibility for bone tissue engineering, the nature of Mg degradation is linked to the evolution of hydrogen gas, which forms gas cavities at the implant–tissue interface [18]. When the local hydrogen concentration is exceeded, hydrogen diffusion from Mg implants can create cavities in tissues [18,19]. Knowledge of the interaction between different cell culture media and photonic interactions is vital for diagnosis and therapeutic applications [20].

In the last decade, many studies investigated the potential for NIRS in bioprocess monitoring, where a range of analytes can be measured in real-time and used for several applications [21,22]. As the fields of NIR spectroscopy and NIR imaging have grown, significant advances have been made in instrumentation, spectral analysis, and applications [23]. This provides molecular information nondestructively, which is one of its most attractive advantages. In addition to molecular studies, NIRS can be used for nondestructive analyses of different materials, such as foods, polymers, and tablets [24,25]. NIRS is used for monitoring the physiological parameters in the body. Because of its low absorption ratio, especially compared to haemoglobin, water, proteins, and collagens, the photons can penetrate deeper (up to a few centimetres) into biological tissues within the NIR optical window (650 nm to 1100 nm) [26]. Although photons above 950 nm are strongly absorbed by water, biological information such as glucose and lactate may be carried in the region between 1000 and 2000 nm [27,28].

This study investigated the Mg degradation and bubble formation behaviour when in contact with DMEM solution using surface measurements and NIRS. DMEM is a common basal medium used to support the growth of numerous different mammalian cells. DMEM is unique compared to other media as it contains four-times higher vitamin concentrations and amino acids from the original Eagle’s medium [29]. The main objective of the proposed study was to present the potential of NIRS to monitor the Mg behaviour changes at different time points, which could also be used in the preclinical trials of Mg in vivo. The surface measurements using scanning electron microscopy (SEM), energy-dispersive X-ray (EDX), profilometry, contact angle measurements, and inductively coupled plasma mass spectrometry (ICP-MS) were examined as standard controls. The comparing of the absorption changes lasted over ten days of immersion. The bubble formation can change the reflectance spectra and pH in vitro. Our null hypothesis was that the continuous physiochemical reaction of Mg-based implants could be optically detected throughout the process in vitro.

## 2. Results

### 2.1. Optical Spectroscopy

**Figure 1:**
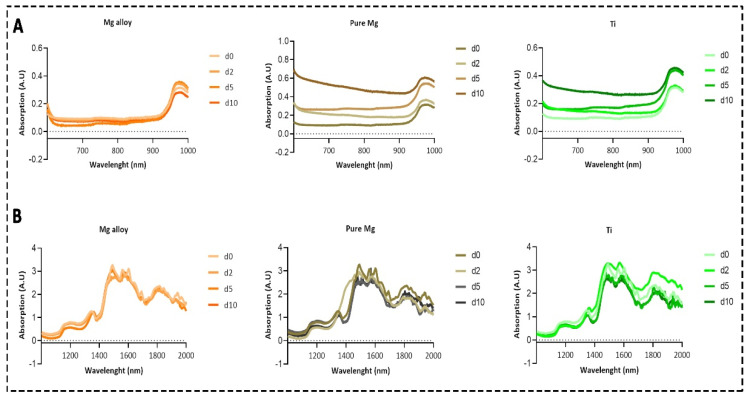
The absorption spectra of DMEM solution containing Mg alloy, pure Mg, and Ti. (**A**) Avantes spectrometer within 600 and 1000 nm; (**B**) results of the NIRQuest Spectrometer from 1000 to 2000 nm.

The absorption of photons that have the same energy as the vibrations of a molecule results in a higher level of excitation of a molecule after it has been irradiated. Overtones and combination bands of fundamental absorptions can be found within the NIR region of the spectrum even though fundamental absorption occurs within the mid-IR region [30]. The absorption spectra of DMEM solution for Mg alloy and pure Mg were changed at each time point due to the chemical reaction in the DMEM solution from the disk surfaces and the formation of Mg bicarbonates and H^+^ ions. Figure 1A shows the spectrum of DMEM solution at four different time points from the 600 to 1000 nm range. The spectra of DMEM solution from the same samples within the 1000 to 2000 nm range are also shown in Figure 1B.

**Figure 2:**
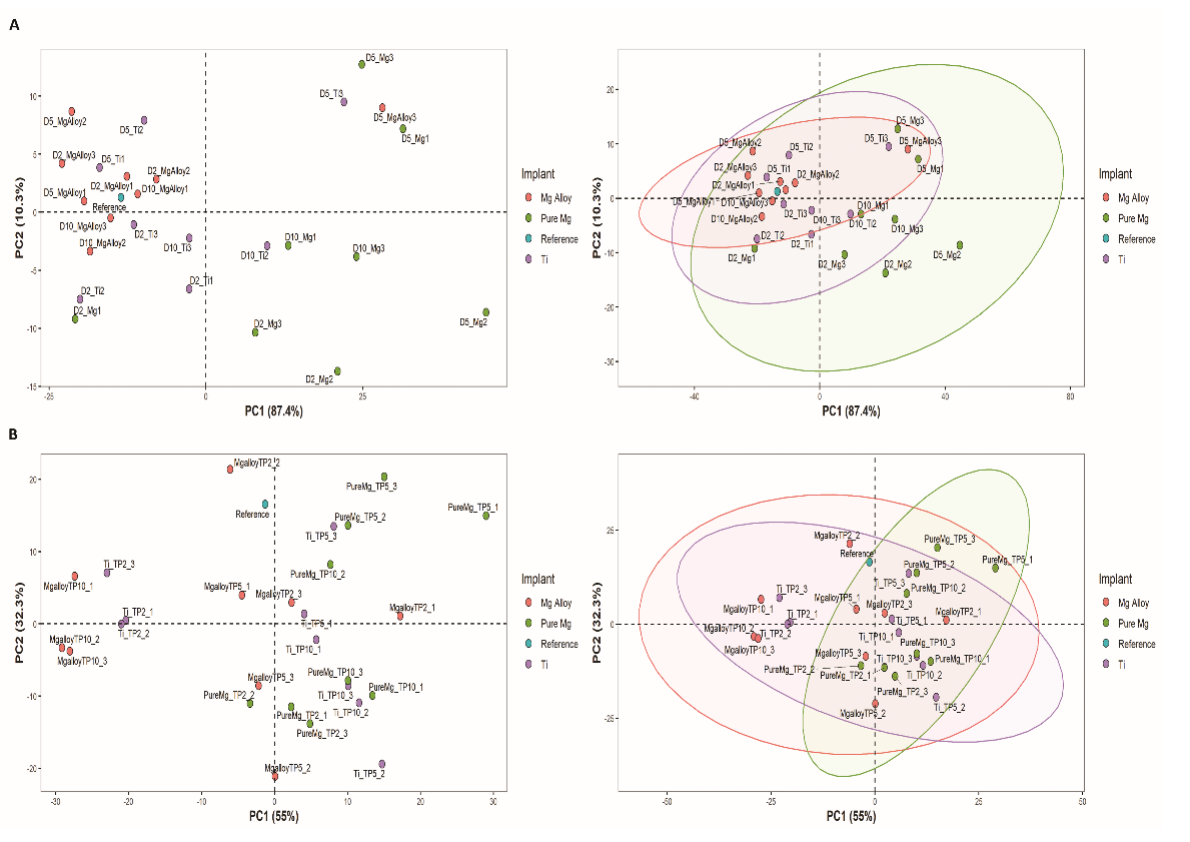
Principal component analysis (PCA) score plots, where samples are coloured by implant type. (**A**) shows the PCA score for absorption spectra ranges from 600 to 1000 nm in wavelength and its corresponding ellipses on the same score plot showing the 95% confidence interval explaining the correlation of the similarity and differences with an increase in the time point; the samples contributed more toward PC1; (**B**) shows the PCA score plot for absorption spectra ranges from 1000 to 2000 nm and its corresponding ellipses on the same score plot showing the 95% confidence interval explaining the correlation of the similarity and differences with an increase in the time point; the samples contributed more toward PC1 except the Mg alloy.

The graphs in Figure 2A,B show the principal component analysis (PCA) score plots and their corresponding ellipses obtained from the absorption of DMEM solution at each time point. The PC1 explains 87.4% of the variance, and PC2 explains 10.3% of the variance, while PC 3 only 1.3%. The confidence ellipses in Figure 2 define the area in which 95% of all samples can be derived from the underlying Gaussian distribution. These confidence ellipses may also be used to visualize correlations between samples. When two variables are uncorrelated, the confidence ellipse becomes more circular.

### 2.2. Surface Analysis

**Figure 3:**
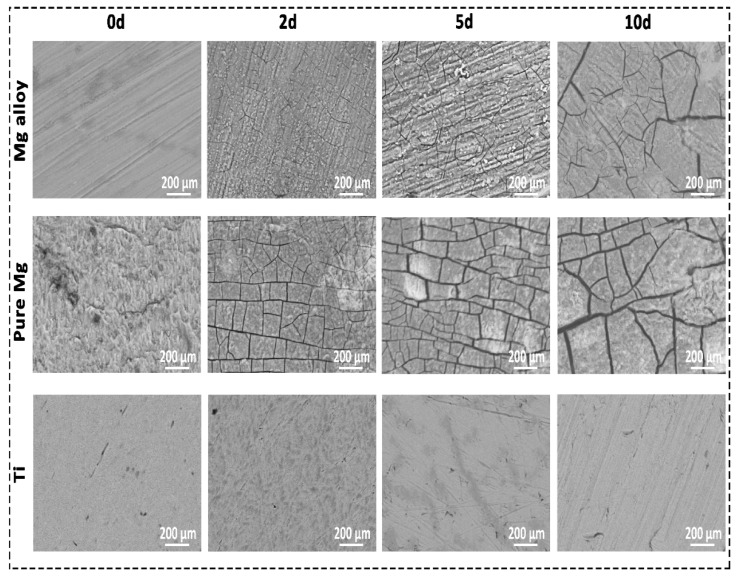
The scanning electron micrograph (SEM) of the surfaces of Mg, pure Mg, and Ti after 0, 3, 5, and 10 days of in vitro corrosion in DMEM solution.

SEM offers detailed high-resolution sample images by rastering a focussed electron beam across the surface and detecting a backscattered signal [31]. EDX was used to provide a quantitative composition and elementary identification information [32]. SEM images of Mg alloy and pure Mg surfaces revealed areas of a deeper corrosion layer in Figure 3. Mg alloy and pure Mg had a specific pattern of degradation that could be detected once the corrosion layer was removed. This was consistent across all solutions, with more severe corrosion visible at longer time intervals and in various solutions with weight loss. However, in the case of Ti, no cracks and corrosion layers were detected even after Day 10. According to preliminary EDX analysis, the superficial corrosion layer on Mg alloy and pure Mg was predominantly formed by calcium (Ca), phosphorus (P), and oxygen (O_2_), whereas the deep layer was primarily Mg and oxygen. For the Ti group, it was mainly Ti (~95 atomic concentration), and a small amount of O_2_ was found (~5 atomic concentration). We observed this pattern in all the Mg samples. Table 1 presents the quantitative composition information for EDX analysis. Complete elemental identification can be found in the Supplementary Material, which includes pure Mg and Ti.

**Figure 4:**
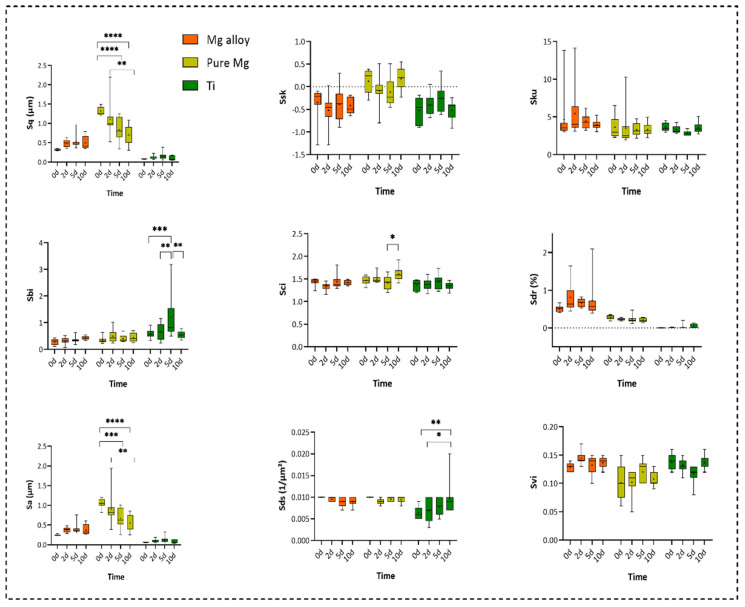
The measurement of surface roughness parameters from Days 0, 2, 5, and 10 including Sq = root-mean-squared height, Ssk = skewness, Sku = kurtosis, Sa = arithmetic mean height, Sbi = surface bearing index, Sci = core fluid retention index, Svi = valley fluid retention index, Sdr = developed interfacial area ratio, and Sds = the summit density (two-way ANOVA post hoc Tukey’s method with statistically significant difference of **** *p* < 0.0001 *** *p* < 0.001, ** *p* < 0.01, * *p* < 0.05 ).

The degradation of the alloy has an impact on the surface topography. This was analysed by means of optical profilometry, and the results are presented as a set of parameters including Sq (root-mean-squared height), Ssk (skewness), Sa (arithmetic mean), Sci (core fluid retention), and Sku (kurtosis), demonstrated in Figure 4. The Sa, Sq, and Sci parameters were significantly higher in pure Mg. However, Sds (the summit density) was significantly higher in Ti. There was no significant difference between the three examined groups regarding other surface parameters.

### 2.3. In Vitro pH Measurement

**Figure 5:**
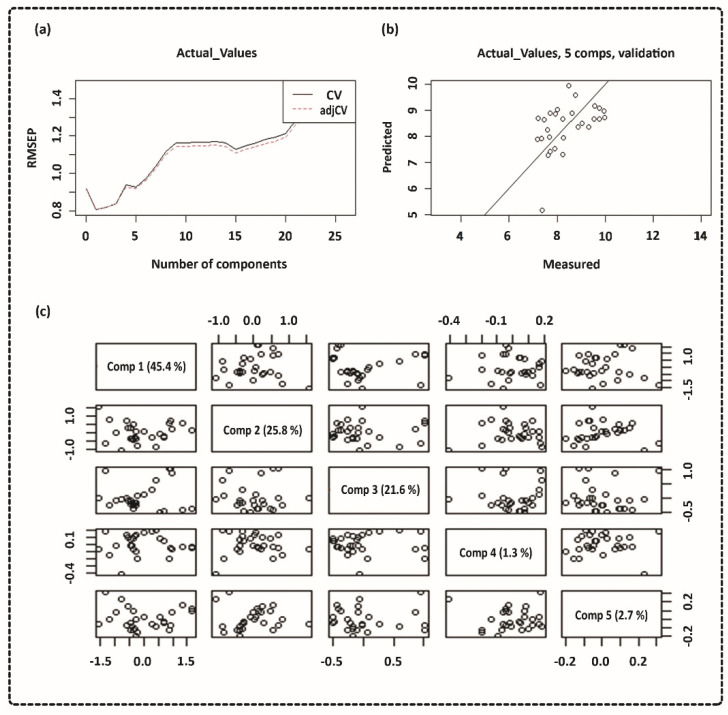
Partial-least-squares regression (PLSR) model using the 1000–2000 nm NIR range. (**a**) Cross-validation of root-mean-squared error of prediction (RMSEP) curves for pH data; (**b**) cross-validation prediction of pH data; (**c**) score plot of pH data.

**Figure 6:**
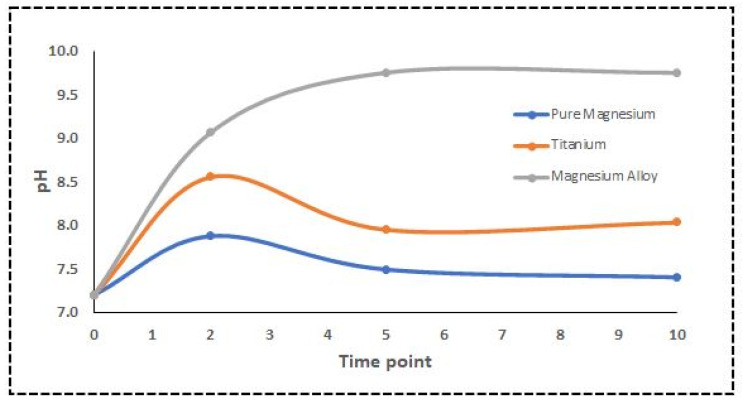
Changes in pH of DMEM solutions containing different disks.

The pH of the DMEM solution significantly influences the corrosion and degradation of the Mg alloy used in biomedical applications [14]. Aliquots of the solution were extracted, and the pH was monitored at each time point. Usually, DMEM solutions’ pH is within 6.8 and 7.2. The measured pH for DMEM containing the Ti samples was almost in the physiological range, while pure Mg was a bit higher than that. However, the pH of the Mg alloy exposed to DMEM was unexpectedly high. NIR spectroscopy and multivariate approaches were used to calculate the pH [33]. Since there is more variation in the mid-NIR spectrum, we used the wavelength range of 1000 to 2000 nm for the PLS model. Our results indicated a substantial NIR prediction of the pH in the DMEM solution using partial least squares (PLS), a multivariate regression model. Finally, the relationship between the actual pH values and spectral data was investigated. Plotting the root-mean-square error of prediction (RMSEP) makes interpreting them much more straightforward. Figure 5 shows the PLS regression model where the CV, predicted, and measured values are plotted along with the principal component regression model. Figure 6 shows the actual pH values measured with a standard pH meter.

### 2.4. Contact Angle Measurement

**Figure 7:**
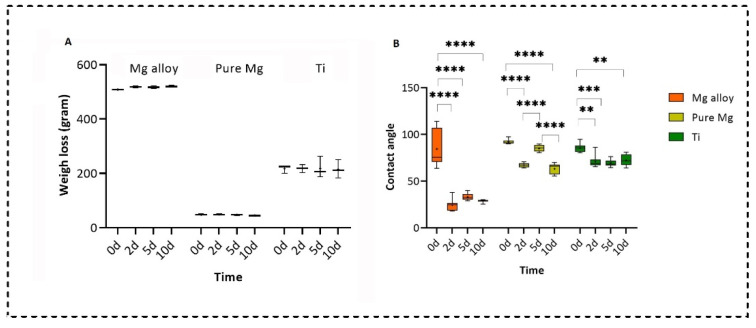
Analysis of (**A**) weight loss and (**B**) contact angle measurements (two-way ANOVA post hoc Tukey method with statistically significant difference of **** *p* < 0.0001, *** *p* < 0.001, ** *p* < 0.01.

We measured the wettability of all three types of samples at different time points using the sessile drop technique by static water contact angle measurements (2 mL, Milli-Q water) [34]. During the contact angle measurement, it was found that the untreated Mg alloys surfaces were hydrophobic, while for pure Mg and Ti, the samples were hydrophilic. After treating the Mg alloy and pure Mg with the DMEM solution, all samples became hydrophilic on Days 2 and 10; however, on Day 5, the samples were hydrophobic. However, no statistically significant difference was found for Ti treatment in the DMEM solution from Day 2 to Day 10. This behaviour was almost similar to the in vivo findings by Rahmati et al. for Mg alloys [35]. Figure 7A,B show the analysis of the weight loss and contact angle measurements, respectively. A significant weight loss was not observed in any of the disks. However, pure Mg showed faster degradation compared to the Mg alloy because pure Mg showed a small amount of weight loss over the Mg alloy’s slight changes.

### 2.5. Distribution of Ion Release in Cell Culture Solution

During the biodegradation process of the pure Mg and Mg alloys, Mg^2+^ and oxidized forms of Mg and its alloys are released. ICP-MS was used to analyse Mg, copper (Cu), manganese (Mn), Zn, silicon (Si), and Ti ions in DMEM solution containing the Mg alloy, pure Mg, and Ti samples. A comparison of these implants was made to thoroughly analyse the impact of the alloys on the accumulation of metal ions in the DMEM samples. Table 2 depicts the results of the ICP-MS studies of Mg alloy, pure Mg and Ti samples in DMEM solution

## 3. Discussion

As part of Mg degradation, electrons are continuously exchanged between anodic and cathodic, explained below (Equations (1) and (2)). As a result, once the OH^−^ and Mg^2+^ saturation limit is reached, the Mg(OH)_2_ is formed in the process [36,37]. Furthermore, Ca ions in the media can produce Ca phosphate products. Because their atomic characteristics are similar, this phosphate layer might be made up of Mg and Ca, which is already shown in the EDX quantification analysis in Table 1 [16]. The degradation behaviour of the sample is greatly affected by the change in the surface composition after soaking in the DMEM solution. Figure 3 shows the SEM morphologies of the Mg alloy, pure Mg, and Ti before and after immersion in the DMEM solution. The DMEM-immersed Mg alloy and pure Mg samples had obvious cracks and flakes of corrosion products on their surfaces. Table 1 shows that the EDX analysis and intensity peaks confirming the existence of Mg, O_2_, Ca, carbon (C), and P on the surface of the Mg alloy. The existence of these elements was also observed in the pure Mg. However, no cracks were observed in the case of Ti. Quantification of the pure Mg and Ti is also available as Supplementary Material in Table S1.

(1) $$\mathrm{Mg}\to {\mathrm{Mg}}^{2+}+2e^{-}$$

(2) $$2H_{2}O\to 2e^{-}=2OH^{-}+H_{2}$$

(3) $$Mg\left(s\right)+2\;H_{2}O\to Mg{\left(OH\right)}_{2}\left(s\right)+H_{2}\left(g\right)$$

ICP-MS analysis of the pure Mg samples revealed a significant increase in Mg^2+^ release compared to the Mg alloy samples, as shown in Table 2. ICP-MS results showed that pure Mg has higher Cu and Mn levels than the Mg alloy and Ti. Even with very low concentrations, the presence of Cu and Mn may be due to Mg degradation and/or surface processing of raw materials [16]. However, in the case of the Si ion, the Mg alloy has a higher concentration for 2d and 5d, while for 10d, pure Mg has a higher concentration level. Furthermore, Table 2 shows that the Mg alloy significantly increases the Zn concentration at each time point compared to the pure Mg and Ti. Zn is considered an essential trace element required for many creatures’ regular metabolism and cellular processes. A recent study showed that Zn accumulation during the degradation process is not toxic [38]. The Mg and Zn concentration in a cell-cultured solution can continue to increase during the immersion period until the alloy has not degraded completely [39]. Finally, ICP-MS showed no toxic trace elements in the cell-cultured solution. Mg corrosion increases the pH values and Mg ion concentrations of immersion solutions in most in vitro experiments, as was observed in the present study by ICP-MS analysis [40].

One of the most fundamental physiological functions of homeostasis is the regulation of body fluid pH because the activity of various chemical processes involving enzyme proteins is heavily dependent on the fluid pH. Alkalinization around the corroded Mg has been shown in various articles to reach pH levels of about ten and even higher [41,42,43]. This process is linked to the formation of OH^−^ and H_2_ due to the water reduction reaction, which supports Mg’s anodic dissolution. During these chemical reactions between the Mg disks and the DMEM solution, OH^−^ was released into the environment, increasing the pH rapidly. In addition, we observed significant changes in the optical properties of the DMEM solution containing the Mg alloy and pure Mg due to continuous chemical reactions, as described in Equations (1)–(3). The main reason behind these significant changes was the degradation of Mg and the following chemical reactions taking place in the DMEM solution. The formation of Mg hydroxide as stated in Equation (3) may occur under various surface roughness conditions. However, it is more likely for an alloy with a smooth surface to produce a continuous protective film than one with an irregular surface [44]. 

We did not observe variations in the Mg alloy’s spectra compared to the pure Mg and Ti within 600 nm to 1000 nm (see Figure 2). However, water absorption was dominant, specifically from 1300 to 1700 nm, influenced by the water reduction reaction, as shown in Equation (2). At each time point, the water dominance increased and decreased due to the chemical reaction, which coincided with the contact angle measurements on these Mg disks. Pure Mg has more variation as compared to the Mg alloy and Ti. The overall trend was in the following order: d5 > d2 > d0 > d10, which corresponded to the in vivo study that was performed on the same Mg alloy [35]. A hydrophobic textured surface has been reported to trap the surface gas layer, protecting against corrosion [45]. Additionally, it may indicate that these gas layers changed the optical spectra in response to the water reduction reaction, influencing the pH changes.

The PCA score plot for wavelength ranges from 1000 to 2000 nm is given in Figure 2B, where more variation is observed in the Mg alloy as compared to the pure Mg and Ti. In a previous study, a tissue-mimicking gel prepared in the biochemistry lab at Oslo Metropolitan University, Norway, was used to investigate the effects of bubble formation in tissues, where we showed that the reflection of NIR light in the Mg alloy sample was 60% higher than the control one [46]. After each time point, disks were placed in the tissue-mimicking phantom to observe the reflection behaviour of bubbles in gels. Next, we selected the concentration of TiO_2_ according to [47], which mimics human breast tissues. In the presence of a hydrogen bubble, higher reflection was observed at each time point, which could be due to scattering in the forward direction. The amount of reflection after Time Point 10 was higher than the control group (no Mg disk).

Specific nutrients must be present in the cell culture solution, such as glucose, sodium pyruvate (energy source), amino acids, sodium bicarbonate, and buffering salts [20]. Coloured substances and pH-dependent dyes are generally included for visual monitoring of any pH changes [20,48]. The pH findings in our study were consistent with the Mg sample corrosion measurements, which showed that high corrosion rates dramatically increased the pH [49,50]. The connection between spectra and actual pH levels was evaluated using PLS modelling, as shown in Figure 5. The water reduction reaction is responsible for these pH variations, as shown in Equations (1) and (2), where electron exchange occurs between anodic and cathodic reactions. When five regression components are used, the validation reaches its closest value, which means that a model with five components would be able to predict the pH value, as shown in Figure 5a. The predicted vs. measured pH for the validated model with five components is presented in Figure 5b. The pH value may be linked to light absorption using PLS analysis, with a correlation coefficient R^2^ of 0.80 and an RMSEP of 0.82. The cross-validated prediction results showed a linear fit, indicating that a model calibrated will predict and estimate the pH levels for this data set. However, some points deviated from the regression line more than others. Figure 5c shows a pairwise plot of the score values for the first five components. A score plot may be used to look for groups, patterns, or outliers in the data. In other words, plotting the data along the first five components informs us about the experiment’s design. Optical spectroscopy has been used to detect pH variations in cultural solution, blood tissue, and pH-induced variations in haemoglobin absorption spectra in both the visible and NIR ranges using the PLS model [51,52,53]. The PLS model used in this study can be further applied to investigate the feasibility of an algorithm that defines the relationship between pH level and spectroscopic data, which also confirms our hypothesis [54]. The spectra obtained from DMEM solutions containing various sample disks were analysed using PCA.

The score plot highlights the sample’s patterns or grouping. This is further depicted in the score plots where distinct colours in the corresponding ellipses represent different samples. Closely grouped samples behave similarly on a score plot, but diametrically opposite samples are negatively correlated [55]. PC1 accounted for most of the variation between the four-set groups. This study shows the potential of surface analysis and optical spectroscopy for in vitro monitoring of Mg degradation, which can be applied in vivo with minimal modification.

## 4. Materials and Methods

We used DMEM as an electrolyte for the experiments, which contains 1 g/L of glucose, sodium bicarbonate, and sterile-filtered, without L-glutamine, ideal for cell culture, imitating body plasma to a certain extent to reproduce the physiological conditions in vitro. Pure Mg, a Mg-based alloy (ZX00, 0.45 wt% Zn-0.45 wt% Ca), and Ti disks (N = 9 per group) were used in this study. Samples were purchased from Britech company. The samples were immersed in the DMEM solution in an incubator (Thermo Forma, Steri-Cycle, Waltham,, MA, USA) containing 5% CO_2_ [14]. The optical spectroscopy method was applied during Days 2, 5, and 10 after immersion. The alloys were kept at 37 degrees in an incubator during the experiment to mimic the body conditions.

### 4.1. Near-Infrared Spectroscopy and pH Estimation

**Figure 8:**
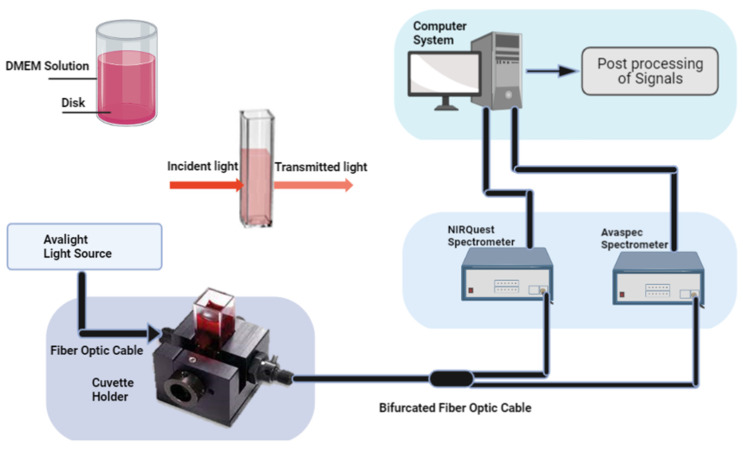
Illustration of NIRS optical setup. A cuvette holder is used to hold the solution for absorption and transmission measurements.

We used NIRS to investigate the cell culture media at four different time points using the dual spectra technique illustrated in Figure 8. For the experimental setup, a halogen lamp (25W) was chosen as a continuous spectrum light source from 300 to 2500 nm, and an Avantes spectrometer (Avaspec 2048x14, BV, Apledoom, the Netherlands) was used to record the spectrum with a 1.2 nm of resolution. An Avantes Spectrometer (Avaspec-2048x14) was used for the 600 to 1000 nm range and a 1000 to 2000 nm Ocean-optics spectrometer (NIRQuest). One other spectrometer, NIRQuest from (Ocean Optics), was used to collect the mid-NIR spectrum. In between the light source and spectrometers, we used a cuvette holder and bifurcated fibre optic cable to measure the absorption and transmission of the DMEM solution. The current sample, reference, and dark data sets in the following equation were used to calculate the absorbance at pixel *n*.

(4) $$A_{n}=\log\left(\frac{sample_{n}-dark_{n}}{ref_{n}-dark_{n}}\right)$$

where *A* is the absorbance, $sample_{n}$ is the analogue to digital counts in pixel *n* when the sample light source is on, $ref_{n}$ is the count in pixel *n* when the reference light source is on, and $dark_{n}$ is the count in pixel *n* without the light source on. An open-source software, R, was used for multivariate analysis of the NIRS data. For the NIR spectral data samples (29x400) and 29 response variables, the actual pH values obtained from the standard pH meter (PHM210, MeterLab^®^, Viroflay, France) were included in the experimental dataset for the PLS model. No additional postprocessing of the data was used except that the spectral data were normalized before applying the PLS regression. The root-mean-squared error of prediction (RMSEP) was used as a marker for validating the results. The PLS model was set for leave-one-out cross-validation (LOO CV). There were two cross-validation estimates: CV and adjCV [54,56]. CV represents the standard cross-validation estimation, while adjCV is the bias-corrected CV estimate.

### 4.2. Scanning Electron Microscopy and Profilometry

Profilometry measurements were performed on an S Neox optical profiler from Sensofar (Spain) controlled with the SensoSCAN 6.7 software, also from Sensofar. Samples were imaged with EPI 20x objective surface parameters obtained from image analysis, and processing was performed using SensoMap Standard 7.4 (Sensofar, Digital Surf’s Mountains Technology^®^, Madrid, Spain). The whole surface was then investigated using SEM and EDX. We used SEM and EDX (Hitachi Analytical TableTop Microscope/Benchtop SEM TM3030) to analyse the surface properties of the samples. Each time point was measured using 1000 × magnification and 15 keV backscatter. EDX spectra were measured for an area of 0.5 mm^2^ in diameter and with a 20 s exposure. The measurements were repeated three times for each sample at each time point, and averages were taken.

### 4.3. Inductively Coupled Plasma Mass Spectrometry

An Agilent 7700 ICPMS/MS (Agilent Technologies, Santa Barbara, California, USA) was used to analyse Cu, Si, Mn, Ti, Zn, and Mg concentrations. For the analyses, the ICPMS/MS was run in He and H2 gas collision mode and no gas mode. All reported concentrations were normalized in relation to the sample mass. The samples were analysed with a 1/10 dilution with HNO_3_ as the conservation fluid. Analyses were performed with two different parameters. No gas mode ICPMS/MS analysis was performed with the following parameters: RF power: 1550 W, RF matching 1.80 Hz, no gas mode, peristaltic pump: 0.1 rps, makeup gas: 0.12 mL/min, and carrier gas: 1.10 L/min. Helium gas collision mode ICPMS/MS analysis was performed with the following parameters: RF power: 1550 W, RF matching 1.80 Hz, peristaltic pump: 0.1 rps, makeup gas: 0.12 mL/min, H2 gas: 6.00 L/min, and He collision gas: 5.0 mL/min. The method used was based on EN ISO17294-1:2007 and EN-ISO17294-2:2016

### 4.4. Statistical Analysis

A Kolmogorov–Smirnov test was used to determine if the dataset’s distributions were parametric or nonparametric. After that, a normality test was performed (Holm–Sidak method). The data are given as arithmetic mean values with the standard deviation when normally distributed; otherwise, they are presented as median values with the interquartile range. When the normality test failed, a two-way ANOVA on ranks was used for post hoc comparison by using the Kruskal–Wallis test. Otherwise, regular ANOVA was used for the rest of the analysis, with a Tukey test for post hoc comparison. GraphPad Prism 8 (GraphPad Software Company, San Diego, CA, USA) was used to conduct all analyses.

## 5. Conclusions

NIRS was used to investigate the degradation behaviour of Mg disks in a cell culture media with multivariate analysis, which was correlated with the ion release information and surface analysis. In addition, the presence of hydrogen bubble formation changes the optical spectrum of the tissue phantom and DMEM solution and also changes the pH, which was predicted by the PLS model with a linear fit, indicating that the pH calculation can be predicted. Furthermore, the surface analysis provided the elemental composition and profilometry results before and after immersion at each time point. It was concluded that corrosion and Mg degradation related to chemical reactions play a role in the physiological environment and can be related to optical measurements. This study demonstrated the potential of NIRS to investigate the degradation behaviour, which is associated with the SEM, EDX, and contact angle measurements, and to try and determine the limitations of using NIRS for analysing Mg degradation. Hence, we used standardized methods for investigating corrosion and corrosion products and investigated whether NIR could reveal any of these parameters. Estimating the pH level using multivariate analysis is one of the potentials of NIRS for evaluating Mg in vitro.

## Figures and Tables

**Table 1:** Quantitative composition and elementary identification information for the Mg alloy in atomic percentage with SD.

	Concentration (Atomic Percentage)			
Element	Day 0, Error (%)	Day 2, Error (%)	Day 5, Error (%)	Day 10, Error (%)
Magnesium	81.41 ± 0.40, 5.1	49.66 ± 4.3, 3.5	22.89 ± 5.60, 1.6	38.4 ± 1.47, 2.2
Oxygen	16.24 ± 0.41, 1.9	42.18 ± 4.05, 4.6	53.74 ± 1.67, 4.8	54.38 ± 2.11, 4.9
Calcium	0.2 ± 0.03, 0	3.30 ± 1.30, 0.2	5.44 ± 1.76, 0.3	4.12 ± 1.29, 0.2
Phosphorus	0	2.67 ± 0.95, 0.2	3.84 ± 1.03, 0.2	2.91 ± 0.79, 0.1
Carbon	0	14.29 ± 2.31, 1.7	13.84 ± 1.89, 1.6	0
Chlorine	0	0	0.25 ± 0.01, 0	0.18 ± 0.06, 0
Zinc	0.1 ± 0.01, 0	0	0	0

**Table 2:** The concentration of ions released was analysed by inductively coupled plasma mass spectrometry (ICP-MS) during the immersion process, showing the concentration of Mg, Cu, Mn, Si, Zn, and Ti ions in parts per million (ppm).

Mg Alloy (ppm)						
Time Point	Mg Ion	Cu Ion	Mn Ion	Si Ion	Zn Ion	Ti Ion
0 d	184	0.0035	0.0047	0.45	0.0145	0.0095
2 d	1140	0.063	0.0038	5.4	1.07	0.0095
5 d	1380	0.017	0.0065	9.3	1.96	0.0095
10 d	2440	0.2	0.007	14.4	4.26	0.024
**Pure Mg (ppm)**						
0 d	183	0.0035	0.0053	0.45	0.0145	0.012
2 d	558	0.111	0.016	4.3	0.037	0.0095
5 d	1830	0.021	0.043	6	0.062	0.0095
10 d	2710	0.034	0.036	31.9	0.091	0.049
**Ti (ppm)**						
0 d	187	0.004	0.0052	0.45	0.0145	0.0095
2 d	182	0.05	0.01	2.5	0.044	0.0095
5 d	183	0.022	0.012	3.8	0.04	0.012
10 d	182	0.015	0.011	6.4	0.022	0.019

## Data Availability

Not applicable.

## Supplementary Materials

The following Supporting Information can be downloaded at: https://www.mdpi.com/article/10.3390/ijms23116099/s1.
