# Supplementary material for: In Vitro Monitoring of Magnesium-Based Implants Degradation by Surface Analysis and Optical Spectroscopy
Source: Int J Mol Sci. 2022 May 29;23(11):6099. doi: 10.3390/ijms23116099 (PMC9181122; doi:10.3390/ijms23116099)
Supplement: Supplementary file 1 [file ijms-23-06099-s001.zip › ijms-1675087-supplementary.pdf]

Table S1: Quantitative composition and elementary identification information for Mg alloy in atomic percentage with SD

Concentration (atomic percentage)				
**Pure Mg**				
**Element**	**Day 0, Error(%)**	**Day 2, Error(%)**	**Day 5, Error(%)**	**Day 10, Error(%)**
Magnesium	74.01± 4.41, 4.2	33.13± 5.69, 1.7	15.44± 2.35, 1.3	16.94± 2.38, 1.2
Oxygen	10.92± 4.91, 1.6	46.47± 3.86, 4.8	61.37± 0.91, 5.9	63.32± 2.47, 6
Carbon	15.05± 2.13, 2.7	16.89± 2.16, 1.7	16.27± 0.32, 2.1	14.31±1.20, 1.8
Calcium	0	1.82± 0.03, 0.2	3.71± 1.47, 0.3	3.07± 0.46, 0.2
Phosphorus	0	1.58± 0.25, 0.2	3.19± 0.26, 0.2	2.35± 0.19, 0.1
**Ti**				
Titanium	100±0, 3	94.29± 4.46, 2.8	86.42±1.55, 2.8	91.48± 2.52, 2.6
Nitrogen	0	3.35± 0.36, 0.3	2.85± 1.32, 0.2	6.22± 1.12, 0.3
Oxygen	0	5.94± 4.5, 1.1	11.86± 0.09, 1.5	2.3± 1.40, 0.8
